# Immune responses in pulmonary sarcoidosis following COVID-19

**DOI:** 10.3389/fimmu.2025.1614461

**Published:** 2025-11-25

**Authors:** Anna Starshinova, Igor Kudryavtsev, Artem Rubinstein, Tatiana Akisheva, Alexey Golovkin, Zoia Korobova, Anastasia Kulpina, Dmitry Kudlay

**Affiliations:** 1 Faculty of Mathematics and Computer Science, Saint Petersburg State University, St. Petersburg, Russia; 2 Department of Faculty Therapy with a Clinic, Almazov National Medical Research Center, St. Petersburg, Russia; 3 Department of Immunology, Institution of Experimental Medicine, St. Petersburg, Russia; 4 Laboratory of Molecular Immunology, Saint Petersburg Pasteur Institute, St. Petersburg, Russia; 5 Department of Pharmacology, Institute of Pharmacy, I.M. Sechenov First Moscow State Medical University, Moscow, Russia; 6 Laboratory of Personalized Medicine and Molecular Immunology, NRC Institute of Immunology FMBA of Russia, Moscow, Russia; 7 Department of Pharmacognosy and Industrial Pharmacy, Faculty of Fundamental Medicine, Lomonosov Moscow State University, Moscow, Russia

**Keywords:** autoimmunity, granulomatous diseases, follicular Th cells, post-COVID-19, pathogenesis, sarcoidosis, Th subsets, Th17

## Abstract

**Background/objectives:**

The complex interplay between sarcoidosis and COVID-19 remains an important area of research, since COVID-19 leads to long-term changes in the immune system. However, COVID-19 is often followed by autoimmune diseases, including newly manifesting sarcoidosis. The goal of this study is to characterize CD4+ T cell subsets, playing a pivotal role in the regulation of innate and adaptive immunity, in the peripheral blood of patients with sarcoidosis after COVID-19.

**Methods:**

The peripheral blood samples from patients with sarcoidosis (n = 61) were studied. We divided patients into two distinct groups: sarcoidosis patients with no history of COVID-19 (n= 30) and COVID-19 convalescent patients with sarcoidosis within 12–24 weeks after recovery (n = 31). Healthy controls (n = 40) were similar in terms of age and sex to patients with sarcoidosis. Immunophenotyping of peripheral blood cells was performed using a ten-color flow cytometry.

**Results:**

Sarcoidosis patients with COVID-19 history had higher levels of T-helper cells (Th) when compared to COVID-19 naïve patients with sarcoidosis, but lower levels when compared to healthy controls. In COVID-19 convalescent patients with sarcoidosis, we noted higher absolute numbers and percentages of CD45RA–CCR7– and CD45RA+CCR7– cells within Th subset. Among COVID-19 convalescent patients with sarcoidosis we also found higher levels of T helper 1 cells and T helper 2 cells (with CXCR5–CCR6–CXCR3+CCR4– and CXCR5–CCR6–CXCR3–CCR4+ phenotypes, respectively) when compared to other groups. We also noted a statistically significant increase in central memory CXCR5+CCR6–CXCR3– follicular Th cells, as wells as effector memory CXCR5+CCR6–CXCR3– and CXCR5+CCR6+CXCR3– follicular Th cells in both groups of patients with sarcoidosis vs. healthy controls.

**Conclusions:**

Our study demonstrated Th cells imbalance in patients with sarcoidosis and COVID-19 history. These findings suggest possible clinical and visual progression of chronic lung sarcoidosis in COVID-19 convalescent patients.

## 1 Introduction

Sarcoidosis is a systemic disease of unknown origin with heterogeneous clinical manifestations, characterized by activation of T cells in the inflammatory sties (1, 2). The disease can be diagnosed at any age and its progression varying from acute to chronic. The most common reaction is seen in thoracic lymphatic nodes and lung tissue, but there are other possible targets, e.g., nervous system, heart, liver, skin tissue (3). As seen in previous research, individuals with genetic predisposition to sarcoidosis with additional triggering factors often develop autoimmune disorders with non-caseating type granulomas in different tissues and organs. Unlike granulomas caused by infection, sarcoidosis does not lead to the formation of necrosis, instead, ATP hyperproduction is quite common (4). Therefore, possible causes for sarcoidosis include pathogens (5), autoimmune inflammation (3), various physical and chemical agents (6), as well as genetic predisposition (7). These factors can affect cells of innate and adaptive immunity.

Since the start of the COVID-19 pandemic, new studies emerge dedicated to sarcoidosis development caused by triggering effects of SARS-CoV-2 (8–11). Despite the unprecedentedly fast development of vaccines against COVID-19 and strong global mass vaccination efforts, the emergence of new variants of SARS-CoV-2 threatens to reverse the progress made in limiting the spread of the disease. Currently, seasonal upsurges of COVID-19 disease are still observed with the detection of different SARS-CoV-2 variants (12). Multiple studies suggest excessive stimulation of systemic inflammation by SARS-CoV-2, followed by tolerance disturbance and abnormal cytokine regulation. These factors lead to granuloma formation. Other authors demonstrate long COVID-19 to be associated with persistent viral reservoirs in tissues and potential reactivation of SARS-CoV-2 (13). SARS-CoV-2 can enter ACE2-expressing cells, but not in those that express other coronavirus receptors, such as aminopeptidase N and dipeptidyl-peptidase 4 (DPP4). These data suggest that ACE2 is a cellular receptor for SARS-CoV-2, explaining why mostly ACE2-expressing cells are affected by SARS-CoV-2 (14). Due to high viral load, immune cells are hyperactivated, which leads to the disruption of their development and functional activity. Severe COVID-19 is often followed by immune cell dysregulation, manifesting in the downsurge of IFNγ-producing Th cells (Th1 and Th17.1), Th17 and Treg levels, with the increase of Th2 levels in peripheral blood (15). Sarcoidosis is also associated with cellular immunity imbalance, followed by B cells, Th2 and Th17 increase with Tregs depletion (16).

Severe central immune system dysfunction has been observed in patients with COVID-19 (15, 17, 18). Thymus dysfunction and impaired ‘naïve’ T cell development can worsen the lymphopenic state in patients with acute COVID-19. This requires more time for qualitative and quantitative regeneration of circulating T cells in the recovery period. Acute COVID-19 is followed by low TREC levels or even their absence in the bloodstream, acting as a marker for unfavorable outcomes. As seen in the paper by Khadzhieva et al., respiratory distress syndrome in COVID-19 is associated with lower TREC concentrations when compared to patients with normal respiratory function (19). Low levels of TREC in COVID-19 patients are described as criteria for lethal outcomes, whereas increased concentrations of TREC molecules are markers of successful recovery (20). Recent studies also demonstrated that human thymus is a potential target for SARS-CoV-2, and thymic function is often impaired after infection (21). Therefore, monitoring thymus functional activity is an important marker for disease severity and progression (21). The results of our previous study also show that thymus dysfunction persists even after recovery (22). Thus, thymus dysfunction can lead to the constriction of the TCR repertoire. This causes to a decrease in ‘naïve’ T cells in peripheral blood and secondary lymphoid organs, leading to insufficient protective immunity (23). Moreover, ‘late’ functional recovery in thymus can contribute to secondary infections following COVID-19 (24). This can affect disease severity (25), and lead to reactivation of other infection, e.g., herpes, in the convalescence period (26). Such prominent changes in T cells maturation in the post COVID-19 period can affect the main condition, i.e., lung sarcoidosis. Moreover, as shown previously, granulomas *de novo* formation in COVID-19 can be a sign of viral initiation of T cellular granulomatous inflammation of autoimmune origin (27).

There are multiple studies on clinical cases of sarcoidosis followed by thymus mal-function with thymoma formation. For example, Tanaka et al. reported malignant thymoma in sarcoidosis, possibly caused by tumor provoking autoimmune sarcoidosis due to defects in T cell tolerance (28). Another study described case includes a patient with pre-existing sarcoidosis and *de novo* thymoma formation (29). Moreover, Esendagli et al. showed thymus’s role in sarcoidosis development and presented a case report about 53 y.o. female with sarcoidosis granulomas in lung tissue, chest lymphatic nodes, and skin underwent thymectomy, which led to regression of sarcoid tissues (30). Hato et al. demonstrated co-existence of the calcified thymoma and sarcoid granulomas, localized in lung tissues and chest lymphatic nodes (31). Moreover, peripheral blood ‘naïve’ CD4+ T cells displayed markers of non-TCR-mediated activation, apoptosis, and differentiation dysregulation, suggesting a persistent inflammatory response of potentially infectious or autoimmune origin (32). Thus, disrupted thymus functions and a decline in ‘naïve’ T cell subpopulations might play an important part in the pathogenesis of sarcoidosis, as well as post-COVID-19 syndrome (21, 27).

Taken together, changes in T-cellular immunity can be expected in patients with sarcoidosis recovering from COVID-19. Furthermore, following acute SARS-CoV-2 infection, various autoimmune diseases have been reported (33, 34), as well as many cases of new onset of sarcoidosis after COVID-19 (reviewed in 27). COVID-19 may affect the clinical course and prognosis of the main condition, mostly due to the fact that patients with sarcoidosis can be more susceptible to autoimmune processes provoked by SARS-CoV-2. Taken together, the levels of CD4+ T cells and their main subsets could be crucial for sarcoidosis and COVID-19 development, prognosis, and outcomes. Therefore, our goal was to characterize dysregulated CD4+ T cell subsets in the peripheral blood of sarcoidosis patients after acute COVID-19.

## 2 Materials and methods

### 2.1 Patients

The peripheral blood samples from patients with sarcoidosis (Sarc) (n = 61) were collected from 2017 to 2023 year (men, n = 36 (59%), women, n = 25 (41%), the average age 36.4 ± 6.2 years) were examined. Patients were first diagnosed with pulmonary and intrathoracic lymph node sarcoidosis (stage II). Patients’ management did not include glucocorticoid therapy. We divided patients into two distinct groups: group I (sarcoidosis patients with no history of COVID-19, Sarc, n = 30) – Sarc (+) COVID-19 (–) history; group II (COVID-19 convalescent patients with sarcoidosis, pC Sarc, n = 31) – Sarc (+) COVID-19 (+) history, blood samples from pC Sarc group were collected 12–24 weeks after recovery from acute SARS-CoV-2 infection. Inclusion criteria: age from 18 to 65 years, presence of verified lung sarcoidosis stage II-III (35, 36) before the acute coronavirus infection. In group II, COVID-19 confirmation was based on positive PCR testing for SARS-CoV-2 RNA, and changes on chest CT. The participants were not previously vaccinated against SARS-CoV-2 due to their medical condition.

Exclusion criteria: sarcoidosis stage IV, history of multi-drug anti-tuberculosis therapy; immunosuppressive therapy at the time of the study; recent course of plasmapheresis; presence of tumor diseases, decompensated diabetes mellitus, and diagnosed autoimmune pathologies.

Healthy controls (HC, n = 40) were in the same ranges of age (34.1 ± 9.7 years) and sex (men – 27, women – 13) as patients with sarcoidosis. The control group included healthy individuals with no history of tuberculosis contacts and anamnesis of sarcoidosis., negative results for the TB blood test (Diaskintest^®^, Generium, Russia), and no pathology on chest X-ray. The controls had no chronic diseases and demonstrated negative results on COVID-19 PCR tests in 2021-2022.

All participants gave their informed consent under guidelines of the Declaration of Helsinki protocol. The study was approved by the local Ethics Committee of Research Institute of Phthisiopulmonology (protocol No. 34.2, 19 January 2017) and the Ethics Committee of the Almazov National Medical Research Centre (Protocol №10-22 dated 03.10.22). The characteristics of patients with sarcoidosis are presented in **Table 1**.

**Table 1:** The characteristics of patients with sarcoidosis.

Characteristics of patients	Sarcoidosis patients with no history of COVID-19, (n = 30)	COVID-19 convalescent patients with sarcoidosis, (n = 31)
Men	20 (67%)	21 (68%)
Women	10 (33%)	10 (32%)
Age	36.5 ± 10.6 years	32.7 ± 6.7 years
Clinical symptoms	27 (90%)	26 (87%)
Fever	20 (67%)	18 (58%)
General weakness	21 (70%)	15 (48%)
Sweating	18 (60%)	8 (26%)
Weight loss	21 (70%)	8 (26%)
Respiratory symptoms		
Cough	22 (73%)	5 (16%)
Shortness of breath	11 (37%)	6 (19%)
Chest pain	5 (17%)	4 (13%)
X-Ray and СT changes		
Enlarged lymph nodes	0	31 (100%)
Lung infiltrates	15 (50%)	5 (16%)
Lung focus	11 (36%)	27 (87%)
Focal infiltrates and focus in the lungs	6 (20%)	3 (10%)
Bacteriology		
Sputum negative for *M.tuberculosis*	30 (100%)	31 (100%)

### 2.2 Methods of the study

The examination of the patients included computed tomography (CT), blood tests, tests for tuberculosis infection (Diaskintest^®^ (Generium, Russia) TB. T-SPOT (Oxford Immunotec Ltd., Abingdon, UK), Mantoux test with 2 TE) (37), morphological examination of the lung and intrathoracic lymph nodes lesions (with transbronchial and videothoracoscopic biopsy). Sarcoid granulomas without caseous necrosis were diagnosed as an accumulation of inflammatory cells: mononuclear cells (monoblasts, promonocytes, monocytes, resident macrophages), some of which with incomplete (imperfect) phagocytosis, CD4+ T cells, CD8+ T cells.

### 2.3 Sample collection

Peripheral blood samples from the patients were collected before the start of the treatment. Five milliliters of peripheral blood were collected from each patient with sarcoidosis and healthy subjects in K3EDTA tubes. Collected peripheral blood samples were processed immediately. CD4+ T cell subsets immunophenotyping was performed within several hours (less than 6 hrs, blood samples were stored at 20-22°C) after blood collection.

### 2.4 Immunophenotyping of peripheral blood CD4+ T cell subset maturation and “polarized” stages

Ten-color flow cytometry was used to analyze the surface phenotype of CD4+ T cell subsets. The list of monoclonal anti-human antibodies is shown in **Supplementary Table 1**. In brief, 200 µL of peripheral blood were stained (at room temperature for 15 min in the dark) with the cocktail of the above-mentioned antibodies according to the manufactory’s recommendations. After antibody staining, red blood cells were lysed (in the dark at room temperature for 15 min) by adding 1.95 mL of VersaLyse Lysing Solution (Beckman Coulter, Inc., Indianapolis, IN, USA) with 50 μL of IOTest 3 Fixative Solution (Beckman Coulter, Inc., Indianapolis, IN, USA). Next, all samples were washed twice (330x g for 8 min) with sterile phosphate-buffered saline (pH 7.4) supplemented with 2% of fetal calf serum (Sigma-Aldrich Co., Saint Louis, MO, USA), resuspended in 500 μL of fresh phosphate-buffered saline (pH 7.4) with 2% neutral formalin (Sigma-Aldrich Co., Saint Louis, MO, USA). All patients’ samples were acquired on a Navios™ 3/10 flow cytometer (Beckman Coulter, Indianapolis, IN, USA), not less then 40 000 Th cells were collected per each sample. Flow-Count™ Fluorospheres (Beckman Coulter, Indianapolis, IN, USA) were used to determine CD4+ T cell subsets absolute numbers (the data were presented as the number of CD4+ T cell subsets per 1 μL of whole peripheral blood).

Gating and analysis strategies for maturation and ‘polarized’ CD4+ T cells were described in details previously (38) and are shown on **Supplementary Figure 1**. In brief, we distinguished CD4+ T cell subset by the expression of CD3 and CD4, as well as by the absence high expression of CD25 and CD8. Next, on the basis of the expression of two surface molecules – CD45RA and CCR7 – CD4+ T cells were divided into four maturation subsets, including ‘naïve’ CD45RA+CCR7+ cells, central and effector memory cells (CD45RA–CCR7+ and CD45RA–CCR7–, respectively), and effector memory CD45RA-positive Th cells (TEMRA, CD45RA+CCR7–). Strategy of flow cytometric analysis for CCR7 and CD45RA expression on the surface of T cell was suggested previously by Sallusto et al. (39).

Currently, there is no commonly used list of markers for the classification of ‘polarized’ Th subsets. However, we based our work on generally accepted recommendations from Maecker et al., 2012 (40), Finak et al., 2016 (41), Brodie et al., 2013 (42), as well as “Guidelines for the use of flow cytometry and cell sorting in immunological studies (second edition)”, published in 2019 (43). In brief, as it is shown on **Supplementary Figure 1**, we used the following anti-chemokine receptors antibodies – CXCR5, CCR6, CXCR3, and CCR4, and we identified main Th cell subsets, including CXCR5+ follicular Th cells (Tfh), CXCR5–CCR6+ Th17 cells, CXCR5–CCR6–CXCR3+CCR4– Th1 cells, and CXCR5–CCR6–CXCR3–CCR4+ Th2 cells. Furthermore, Th17 cells were subdivided into ‘classical’ CCR6+CCR4+ Th17 cells, ‘non-classical’ CCR6+CXCR3+ Th17.1, and lacking (‘double negative’ or DN Th17) or co-expressing CXCR3 and CCR4 (‘double positive’ or DP Th17) Th17 cells. While CXCR5+ Tfh cell were subdivided into Tfh1 (CXCR3+CCR6–), Tfh17 (CXCR3–CCR6+), Tfh2 (CXCR3–CCR6–), and ‘double positive’ DP Tfh (CXCR3+CCR6+). All mentioned Th cell subsets were identified within total CD4+ T cells (the data were presented as the % within total CD4+ T cell subsets and in absolute numbers – the number of cells per 1μL pf whole peripheral blood), as well as within central and effector memory cells with CD45RA–CCR7+ and CD45RA–CCR7– phenotypes, respectively.

### 2.5 Statistical analysis

Kaluza™ software v2.3 (Beckman Coulter, Indianapolis, IN, USA; www.beckman.com) was used for flow cytometry data analysis. Flow cytometry data of HCs and patients with sarcoidosis were concatenated and analyzed using the t-Distributed Stochastic Neighbor Embedding (tSNE) native platform in FlowJo™ v10 Software (BD Bioscience Inc., Ashland, OR, USA; www.flowjo.com). Considering that the objects of interest were small subpopulations of lymphocytes, tSNE was chosen as a more sensitive dimensionality reduction method to the local structure of the data. Data were analyzed using GraphPad Prism 8 (GraphPad software Inc., San Diego, CA, USA; www.graphpad.com) and Statistica 7.0 (StatSoft, Tulsa, OK, USA; www.statistica.software.informer.com/7.0/) software packages. The obtained data were tested for normality of distribution via the Shapiro-Wilk test. According to Shapiro-Wilk test (p < 0.05) the data on T cell subsets from all groups of patients and HC significantly deviate from a normal distribution. Thus, the statistical comparison of data between patients with sarcoidosis, COVID-19 convalescent patients with sarcoidosis and healthy controls were performed using the non-parametric Kruskal Wallis test with *post-hoc* Dunn’s test. The differences between the groups were estimated using Mann–Whitney U-test. Differences were considered statistically significant with p < 0.05 value.

### 2.6 Declarations ethical approval

The study was conducted according to the guidelines of the Declaration of Helsinki, and approved by the local Ethics Committee of Research Institute of Phthisiopulmonology (protocol No. 34.2, 19 January 2017) and the Ethics Committee of the Almazov National Medical Research Centre (Protocol №10-22 dated 03.10.22). All patients included in the study provided informed consent.

## 3 Results

### 3.1 Alteration in main CD3+ T cell subsets in patients with sarcoidosis and COVID-19 convalescent patients with sarcoidosis

**Figure 1:**
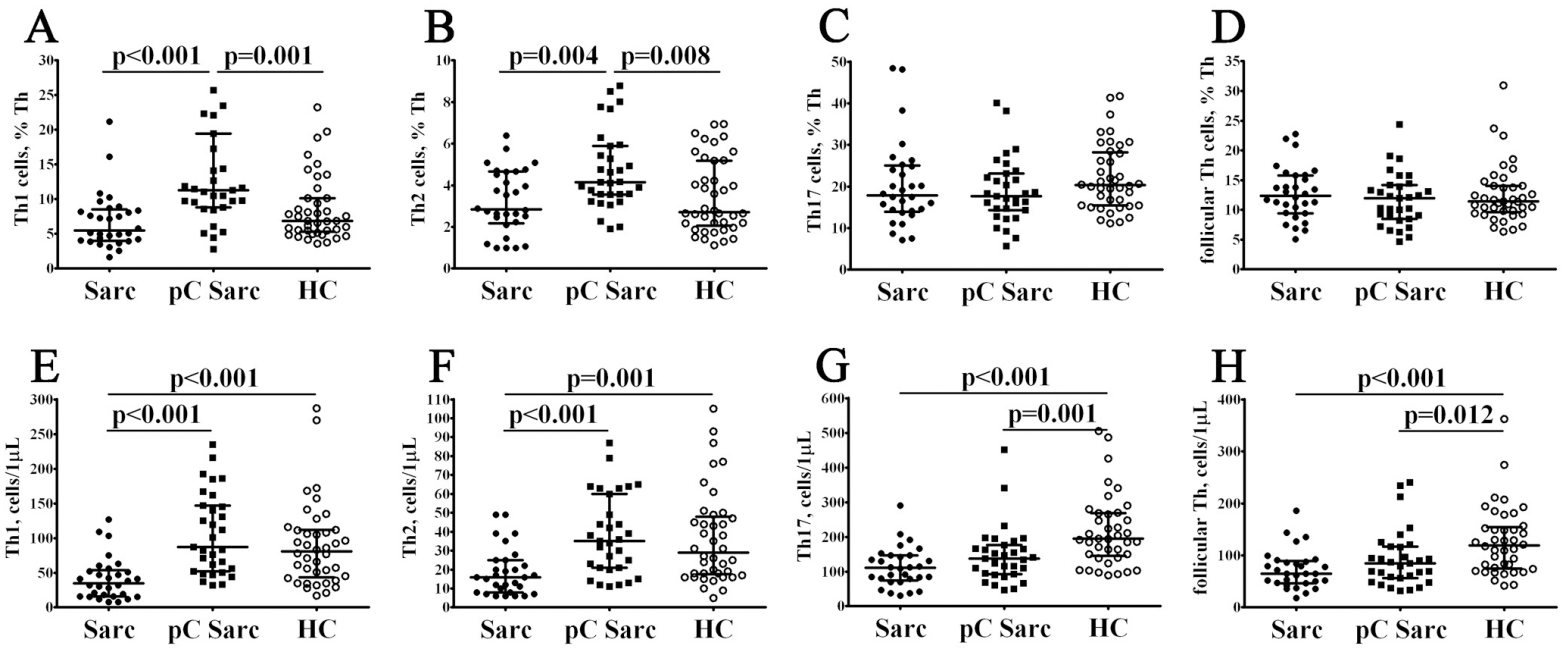
Alteration in main CD3+ T cell subsets in patients with sarcoidosis and COVID-19 convalescent patients with sarcoidosis. Scatter plots **(A–D)** and scatter plots **(E–H)** showing the percentages and absolute numbers of CD3+, CD3+CD4+, CD3+CD8+, and regulatory T cells (Tregs), respectively. Note for **Figures 1**-**4**: Black circles denote patients with sarcoidosis without SARS-CoV-2 infection (Sarc, n = 30); black squares – COVID-19 convalescent patients with sarcoidosis (pC Sarc, n = 31); white circles – healthy control (HC, n = 40). Each dot represents individual subjects, and horizontal lines represent the median of the distribution, whiskers represent the 25% and 75% quartiles [Med (Q25; Q75)]. The statistical analysis was performed with Kruskal Wallis test with *post-hoc* Dunn’s test. The differences between the groups were estimated using Mann–Whitney U-test.

Primarily, we noticed the absolute numbers of CD3+ cell were decreased patients with sarcoidosis (Sarc) when compared to COVID-19 convalescent patients with sarcoidosis (pC Sarc) and healthy controls (964 cells/1μL (724; 1153) vs. 1360 cells/1μL (1080; 1578) and 1595 cells/1μL (1181; 2029) with p = 0.001 and p < 0.001, respectively) (**Figure 1**). Furthermore, frequencies of circulating CD3+ were significantly lower in pC Sarc then in HC group (70.41% (64.26; 77.01) vs. 73.48% (69.55; 78.93) with p = 0.023 and 1360 cells/1μL (1080; 1578) vs. 1595 cells/1μL (1181; 2029) with p = 0.042). Next, the percentages of Th cells were significantly lower in Sarc and pC Sarc groups when compared to controls (40.56% (32.72; 46.24) and 38.50% (33.23; 48.30) vs. 48.18% (42.85; 51.70) with p = 0.009 and p < 0.001, respectively). Moreover, patients with sarcoidosis presented sustained lower counts of CD4+ T cells than pC Sarc and HC groups (551 cells/1μL (451; 762) vs. 747 cells/1μL (569; 960) and 945 cells/1μL (691; 1321) with p = 0.008 and p < 0.001, respectively). Furthermore, pC Sarc patients also showed decreased numbers of CD8+ T cells (p = 0.015).

Overall, the relative numbers of CD8+ T cells was assessed in Sarc, pC Sarc and HC individuals, and no significant differences among groups were detected. But we noticed that CD8+ T cell concentration was decreased in Sarc vs. pC Sarc and HC group (335 cells/1μL (222; 454) vs. 421 cells/1μL (302; 604) and 529 cells/1μL (373; 664) with p = 0.011 and p = 0.001, respectively). Finally, pC Sarc patients had significantly higher frequencies of CD3+CD4+CD25bright regulatory T cells (Tregs) compared to Sarc and HC groups (4.38% (3.80; 5.23) vs. 3.10% (2.12; 3.91) and 3.10% (2.62; 3.58) with p < 0.001 in both cases, and 82 cells/1μL (66; 118) vs. 39 cells/1μL (22; 57) and 67 cells/1μL (51; 86) with p < 0.001 and p = 0.031, respectively), while Sarc patients showed lower absolute numbers of Tregs vs. HCs (p < 0.001).

### 3.2 Alterations in frequencies of circulating CD4+ T cell maturation subsets in patients with sarcoidosis and COVID-19 convalescent patients with sarcoidosis

**Figure 2:**
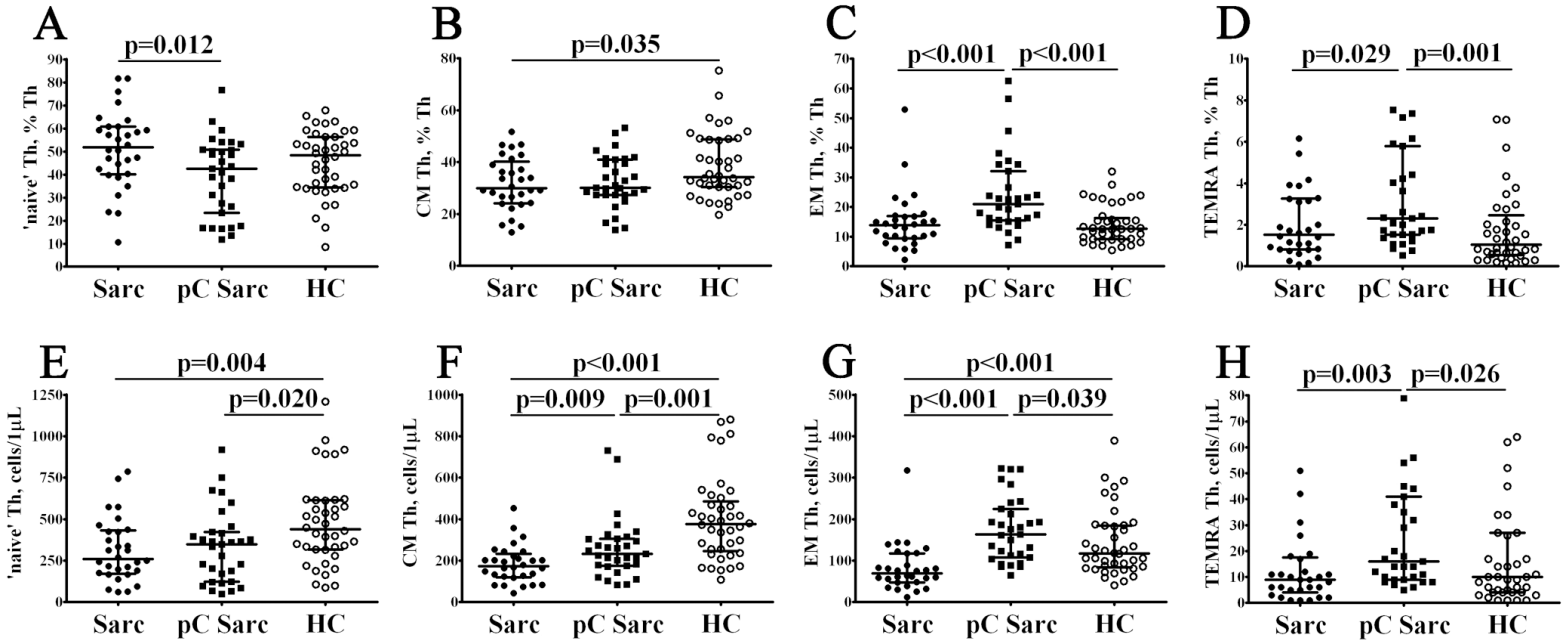
Alterations in frequencies of circulating CD4+ T cell maturation subsets in patients with sarcoidosis and COVID-19 convalescent patients with sarcoidosis. Scatter plots **(A–D)** and scatter plots **(E–H)** showing the percentages and absolute numbers of ‘naïve’, central memory, effector memory and effector memory Th cells re-expressing CD45RA cells (TEMRA) Th cells, respectively.

Next, to examine the frequencies and distribution of CD4+ T cell maturation subsets in different groups of patients with sarcoidosis, we analyzed CD45RA and CCR7 co-expression on Th cells (**Supplementary Figure 1**). When studying CD4+ T cell maturation (**Figure 2**; **Supplementary Figure 2**), we noted that pC Sarc group had significantly lower percentages of ‘naïve’ Th cells then Sarc group (42.47% (23.31; 50.75) vs. 51.74% (40.30; 60.71), p = 0.012). Levels of EM and TEMRA Th cell were elevated when compared to Sarc group (20.97% (15.45; 32.18) vs. 13.92% (9.58; 16.81) and 2.31% (1.53; 5.79) vs. 1.53% (0.82; 3.24) with p < 0.001 and p = 0.029, respectively), as well as with HC group (20.97% (15.45; 32.18) vs. 12.62% (9.17; 16.35) and 2.31% (1.53; 5.79) vs. 1.04% (0.55; 2.46) with p < 0.001 and p = 0.001, respectively). Next, pC Sarc patients presented higher counts of CM, EM and TEMRA Th cells then Sarc group (233 cells/1μL (176; 306) vs. 174 cells/1μL (123; 222) with p = 0.009; 163 cells/1μL (108; 225) vs. 69 cells/1μL (48; 117) with p < 0.001; and 16 cells/1μL (9; 41) vs. 9 cells/1μL (5; 17) with p = 0.003, respectively). Finally, we observed a significant reduction of absolute numbers in both ‘naïve’ and CM Th cells (348 cells/1μL (123; 423) vs. 439 cells/1μL (317; 614) with p = 0.020 and 233 cells/1μL (176; 306) vs. 377 cells/1μL (246; 485) with p = 0.001, respectively), while EM and TEMRA Th cells showed significant increase in pC Sarc vs. HCs (163 cells/1μL (108; 225) vs. 117 cells/1μL (84; 185) with p = 0.039 and 16 cells/1μL (9; 41) vs. 10 cells/1μL (4; 27) with p = 0.026, respectively). Thus, COVID-19 convalescent patients displayed higher frequencies (both in relative and absolute numbers) of most mature Th subsets (EM and TEMRA), when compared to sarcoidosis patients with no history of COVID-19.

### 3.3 Alterations in frequencies of ‘polarized’ CD4+ T cell subsets in patients with sarcoidosis and COVID-19 convalescent patients with sarcoidosis

**Figure 3:**
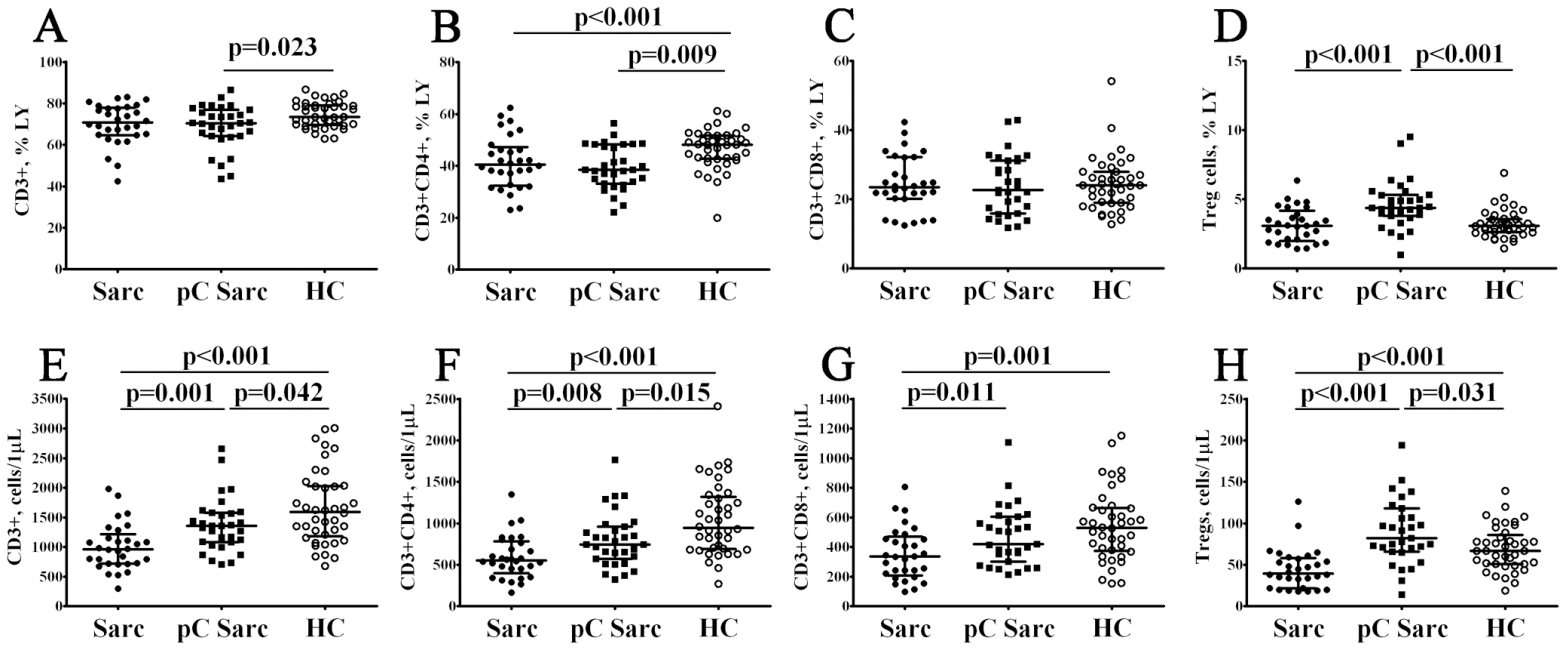
Alterations in frequencies of ‘polarized’ CD4+ T cell subsets in patients with sarcoidosis and COVID-19 convalescent patients with sarcoidosis. Scatter plots **(A–D)** and scatter plots **(E–H)** showing the percentages and absolute numbers of Th1 cells, Th2 cells, Th17 cells, and follicular Th cells, respectively.

To further investigate Th responses, we applied CXCR5, CCR6, CXCR3 and CCR4 chemokine receptor-based gating strategy to identify main polarized Th subsets, as it was described previously (38). Primarily, total conventional (after the exclusion of Tregs from our flow cytometric analysis) CD3+CD4+ Th cell compartment was categorized into 4 functional subsets, including Th1, Th2, Th17 and follicular Th cells (Tfh) (**Supplementary Figure 1**). We found (**Figure 3**; **Supplementary Figure 3**) that pC Sarc presented higher percentages of Th1 then Sarc or HC groups (11.30% (8.82; 19.46) vs. 5.48% (4.03; 8.37) with p < 0.001 and 6.88% (5.32; 10.15) with p = 0.001, respectively). Next, pC Sarc patients maintained significantly higher absolute numbers of Th1 cells compared to Sarc group (87 cells/1μL (52; 147) vs. 35 cells/1μL (16; 52), p < 0.001). When interrogating Th2 cell frequencies, we observed a significant increment in both the relative and absolute levels of Th2 cells in pC Sarc group if compared to Sarc group (4.15% (3.55; 5.89) vs. 2.84% (2.25; 4.65) with p = 0.004 and 35 cells/1μL (21; 60) vs. 16 cells/1μL (8; 25) with p < 0.001). We observed that while the proportion of Th17 and Tfh cells was not significantly different between groups, there were decreased absolute numbers of Th17 and Tfh cells in both of patients with sarcoidosis vs. HCs (110 cells/1μL (79; 1450 and 137 cells/1μL (93; 176) vs. 195 cells/1μL (145; 268) with p < 0.001 and p = 0.001 for Th17, respectively, and 65 cells/1μL (47; 88) and 85 cells/1μL (56; 117) vs. 120 cells/1μL (75; 155) with p < 0.001 and p = 0.012, respectively, for follicular Th cells). Collectively, our data suggest a widely altered spectrum of peripheral ‘polarized’ CD4+ T cells in pC Sarc patients vs. Sarc patients.

### 3.4 Imbalance of ‘polarized’ Th cell subsets within CM and EM Th cell compartments in patients with sarcoidosis and COVID-19 convalescent patients with sarcoidosis

**Figure 4:**
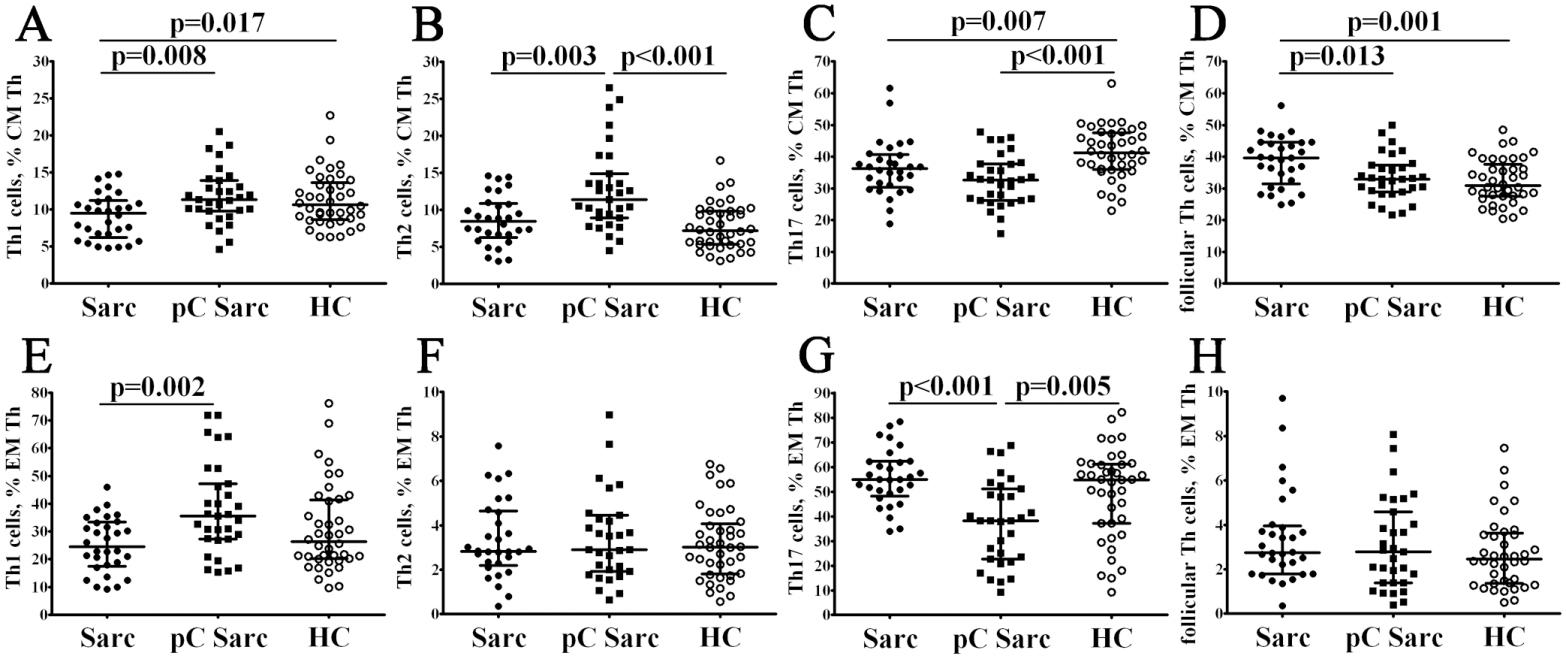
Imbalance of ‘polarized’ Th cell subsets within CM and EM Th cell compartments in patients with sarcoidosis and COVID-19 convalescent patients with sarcoidosis. Scatter plots **(A–D)** and scatter plots **(E–H)** showing the percentages central and effector memory Th1 cells, Th2 cells, Th17 cells, and follicular Th cells, respectively.

To assess the potential effects of COVID-19 on the peripheral blood CM and EM Th cell subsets, we analyzed main ‘polarized’ Th subsets within CD45RA–CCR7+ and CD45RA–CCR7– Th cells, that recirculate through blood, lymphatic system and secondary lymphoid organs, or migrate to inflamed peripheral tissues (44). Primarily, we observed a significant increase of Th1 cells within CM and EM Th cells in pC Sarc patients vs. Sarc patients (11.34% (9.81; 13.96) vs. 9.52% (6.67; 11.06) with p = 0.008 and 35.50% (27.26; 47.20) vs. 24.44% (17.91; 33.06) with p = 0.002, respectively) (**Figure 4**; **Supplementary FigureD 4**, **5**). Next, within CM Th compartment Th2 cells were significantly increased in pC Sarc group vs. Sarc and HC groups (11.38% (8.96; 14.94) vs. 8.46% (6.69; 10.79) and 7.21% (5.37; 9.87) with p = 0.002 and p < 0.001, respectively). Furthermore, we found that frequencies of CM and EM Th17 cells were significantly reduced in pC Sarc patients vs. HCs (32.72% (26.22; 37.79) vs. 41.21% (36.01; 47.55) with p < 0.001 and 38.20% (22.65; 51.20) vs. 54.79% (37.18; 61.13) with p = 0.005, respectively). Similarly, within EM Th cells the proportion of Th17 cells was reduced in pC Sarc in comparison with Sarc (38.20% (22.65; 51.20) vs. 54.87% (49.18; 62.21) with p < 0.001). Finally, the frequency of CM CXCR5+ Tfh cells was significantly decreased in pC Sarc patients vs. Sarc patients (32.98% (28.92; 37.31) vs. 39.66% (32.48; 4.48) with p = 0.013).

### 3.5 The frequency of Th17 cell subsets was not substantially different among patients with sarcoidosis and COVID-19 convalescent patients with sarcoidosis

Next, we assessed CCR4 and CXCR3 co-expression to further study the differentiation of Th17 cells, and we identified ‘classical’ Th17 cells, ‘non-classical’ Th17.1, DN Th17 and DP Th17 cells (50). First, we analyzed the frequency of Th17 cell subsets within CM and EM Th17 cells (**Table 2**; **Supplementary Figure 6**) and found that the levels of all four types of Th17 cell were similar in two groups of patients with sarcoidosis. But, when we compared the frequencies of ‘classical’ Th17 cell in CM Th17 cell, we noticed that the level of these cell was decreased in both groups of patients with sarcoidosis vs. HCs (**Table 2**). Furthermore, the frequencies of CM Th17.1 cells were decreased in both groups of patients with sarcoidosis vs. HCs. Moreover, EM Th17.1 were significantly decreased only in pC Sarc patients vs. HCs. Finally, Sarc patients displayed higher levels of CM DP Th17 and EM DP Th17 cells vs. healthy.

**Table 2:** Central and effector memory Th17 cell subsets in patients with sarcoidosis and COVID-19 convalescent patients with sarcoidosis.

Th17 subset	Sarcoidosis (n = 30)	Sarcoidosis after COVID-19 (n = 31)	Healthy control (n = 40)	Significant Differences
% of cells within total CM Th17 subset:				
DN Th17	11.47 (9.68; 13.76)	13.47 (9.97; 21.48)	11.72 (9.12; 15.33)	p1 = 0.081 p2 = 0.953 p3 = 0.080
‘classical’ Th17	27.33 (19.78; 32.18)	26.24 (19.95; 30.20)	19.63 (16.90; 24.29)	p1 = 0.840 p2 = 0.002 p3 = 0.001
Th17.1	42.94 (38.58; 53.45)	43.74 (34.82; 50.60)	53.31 (46.80; 60.04)	p1 = 0.471 p2 = 0.001 p3 < 0.001
DP Th17	16.84 (11.97; 18.32)	11.85 (8.42; 18.00)	11.17 (8.77; 15.14)	p1 = 0.094 p2 = 0.011 p3 = 0.880
% of cells within total EM Th17 subset:				
DN Th17	5.39 (3.50; 11.79)	6.69 (5.49; 9.45)	5.72 (4.25; 7.50)	p1 = 0.292 p2 = 0.877 p3 = 0.086
‘classical’ Th17	15.40 (11.57; 23.15)	19.01 (14.73; 26.93)	16.92 (12.20; 22.87)	p1 = 0.063 p2 = 0.618 p3 = 0.151
Th17.1	55.27 (48.61; 67.10)	52.05 (41.95; 63.70)	61.76 (53.63; 66.75)	p1 = 0.334 p2 = 0.138 p3 = 0.016
DP Th17	18.55 (12.99; 23.30)	17.52 (10.13; 22.34)	13.41 (9.93; 19.35)	p1 = 0.594 p2 = 0.017 p3 = 0.120

Note for **Tables 2**, **3**: p1—statistical differences between patients with sarcoidosis and COVID-19 convalescent patients with sarcoidosis; p2—statistical differences between patients with sarcoidosis and healthy controls; p3—statistical differences between COVID-19 convalescent patients with sarcoidosis and healthy controls. The statistical analysis was performed with Kruskal Wallis test with *post-hoc* Dunn’s test. The differences between the groups were estimated using Mann–Whitney U-test.

The quantitative data (% of Th17 cell subsets within CM and EM CXCR5–CCR6+ Th17 cells) are presented as median and quartile ranges (Med (Q25; Q75).

### 3.6 Alterations in central and effector memory follicular Th cell subsets in patients with sarcoidosis and COVID-19 convalescent patients with sarcoidosis

Co-expression of CXCR3 and CCR6 chemokine receptors is used to determine the four subtypes of follicular Th cells, including Tfh1, Tfh17, Tfh2, and ‘double positive’ or DP Tfh (53). We observed that the proportions of Tfh1, Tfh2 and Tfh17 cell subsets were not significantly different between groups of patients with sarcoidosis in both the CM and EM Th17 cells (**Table 3**, **Supplementary Figure 7**). In contrast, the levels of CM and EM DP Tfh were reduced in pC Sarc patients vs. Sarc patients. Finally, both groups of patients with sarcoidosis showed increased levels of CM Tfh2 and decreased frequencies of Tfh17.1 of CM DP Tfh if compared to healthy controls. In contrast, the frequencies of Tfh1 within EM CXCR5+ CD4+ T cells were significantly lower in both groups of patients with sarcoidosis compared and healthy controls, while Tfh2 and Tfh17 cells were significantly elevated (**Table 3**).

**Table 3:** Alterations in central and effector memory follicular Th cell subsets in patients with sarcoidosis and COVID-19 convalescent patients with sarcoidosis.

Follicular Th subset	Sarcoidosis (n = 30)	Sarcoidosis after COVID-19 (n = 31)	Healthy control (n = 40)	Significant Differences
% of cells within total CM follicular Th subset:				
Tfh1	28.74 (21.76; 32.64)	30.59 (24.36; 34.73)	30.92 (24.67; 36.56)	p1 = 0.237 p2 = 0.112 p3 = 0.817
Tfh2	20.24 (18.02; 23.76)	23.70 (20.04; 25.78)	16.96 (14.23; 21.65)	p1 = 0.051 p2 = 0.012 p3 < 0.001
Tfh17	34.54 (31.00; 40.69)	30.80 (25.77; 40.93)	33.10 (28.04; 38.77)	p1 = 0.166 p2 = 0.208 p3 = 0.899
DP Tfh	15.64 (11.60; 17.75)	11.45 (9.06; 14.98)	17.92 (13.81; 22.29)	p1 = 0.002 p2 = 0.035 p3 < 0.001
% of cells within total EM follicular Th subset:				
Tfh1	57.02 (40.55; 65.54)	57.93 (50.72; 65.39)	68.61 (58.02; 73.53)	p1 = 0.436 p2 < 0.001 p3 = 0.010
Tfh2	19.11 (13.64; 23.81)	20.00 (13.97; 25.71)	14.10 (9.82; 20.99)	p1 = 0.697 p2 = 0.023 p3 = 0.010
Tfh17	15.27 (9.33; 19.59)	13.77 (10.48; 18.85)	8.56 (6.29; 13.66)	p1 = 0.784 p2 = 0.006 p3 = 0.008
DP Tfh	10.48 (5.19; 13.81)	6.59 (4.38; 9.41)	6.54 (4.52; 11.56)	p1 = 0.019 p2 = 0.138 p3 = 0.711

The quantitative data (% of Tfh cell subsets within CM and EM CXCR5+ Tfh cells) are presented as median and quartile ranges (Med (Q25; Q75).

## 4 Discussion

To our knowledge, this study is the first one to describe alterations in circulating CD4+ T cell subsets in COVID-19 convalescent patients with sarcoidosis. Moreover, we noted significant changes in CD4+ T cell maturation and ‘polarization’ subsets. COVID-19 seemingly can affect not only T cell differentiation within the thymus but also affect CD4+ T cell development in peripheral lymphoid organs.

CD3+ T cell subsets play the central part in adaptive immune response. T-helper cells (CD4+ T cells) stimulate three types of cell-mediated effector immunity and humoral adaptive immunity (44). Cytotoxic T lymphocytes (CD8+ T cells) play crucial roles in immune defense against infections and cancer by direct killing of target cells and cytokine production (45). Regulatory T cells (Tregs) suppress all types of immune responses and maintain tolerance to self-antigens (46). We noticed that the percentage of CD4+ T cells was reduced in the peripheral blood of pC Sarc patients (independently of disease severity and presence of protective antibodies). Conversely, CD8+ T cells were significantly increased, and we also observed an increase in CD8+ T cells in pC Sarc patients to COVID-19 ‘naïve’ sarcoidosis patients (**Figure 1**). While CD4+ and CD8+ lymphopenia has been documented in patients with sarcoidosis, it is not a universal clinical hallmark of sarcoidosis. Sarcoidosis is a highly dynamic process involving multiple immune cell subsets (47). Given the compartmentalized nature of sarcoidosis, it is important to note that peripheral T cell phenotypes may not fully reflect immunologic events in the lung parenchyma or lymphoid tissues. Future investigations should include BALF or granulomatous tissue analyses to clarify the local effects of SARS-CoV-2 on pulmonary immune dynamics in sarcoidosis.

Upon antigen activation in secondary lymphoid tissues, thymus-divided CD45RA+CCR7+ ‘naïve’ CD4+ T cells differentiate into effector and memory subsets (44). Memory cells are characterized by clonal expansion and enhanced ability to respond to previously recognized antigens. Peripheral blood circulating memory cells can be located in the secondary lymphoid organs (CD45RA–CCR7+ central memory cells) or in the peripheral tissues – effector memory cells (CD45RA–CCR7– EM cells) (39). Central memory Th cells are responsible for the long‐term maintenance of immunological memory and exhibit enhanced proliferation potential, while EM Th cells provide rapid cytokine production upon antigen recognition, but have limited ability to proliferate (48). CD45RA–CCR7– TEMRA cells are the most effector cells in circulation. They are short-lived, and exhibit low proliferative activity, lose expression of co-stimulatory molecules and shorten their telomeres (49). We observed that in pC Sarc patients the frequencies of EM and TEMRA CD3+CD4+ cells were increased, both compared to HCs and Sarc groups (**Figure 2**; **Supplementary Figure 2**). The percentage of ‘naive’ CD4+ T cells was decreased compared to patients without COVID-19; their absolute numbers were also lower than in the control group, suggesting reduced thymic output. This may be directly related to an increased risk of infections and/or malignancies, e.g., due to a diminished immune response to new antigens, as well as an increased risk of autoimmunity, potentially linked to decreased numbers of thymus-derived regulatory T cells and impaired immune tolerance to self-antigens. Previous studies noted that patients with sarcoidosis have significantly elevated levels of memory CD4+CD45R0+ Th cells compared to controls (50), although other research did not observe these changes in peripheral blood (51). Our data showed that the frequencies of CM and EM Th cells were higher in pC Sarc group compared with COVID-19 ‘naïve’ patients with sarcoidosis, although the role of these Th cell subsets in the development and progression of sarcoidosis is still unclear.

Th1 cells play the main part in type 1 immune responses against intracellular pathogens such as mycobacterial species and viruses (52). While, Th2 cells participate in type 2 immune responses to extracellular pathogens such as helminthes and their toxins. Th17 cells stimulate the type 3 immune responses to extracellular pathogens including some bacteria and fungi. Finally, Tfh cells help B cells in antibody production and affinity maturation. Furthermore, Th1, Th17 and follicular Th cells are involved in the pathogenesis of autoimmune disorders, while Th2 cells can cause allergic diseases (52). Sarcoidosis development has long been associated with Th1 cells (53), but more recent studies were focused on the key role of Th1/Th17 balance in granuloma formation (54). During acute COVID-19, Th1 and Th17 cells also play multiple roles in both lung tissue damage via destruction of infected cells, and tissue repair. Th1 migration may be confirmed by decrease of Th1 percentage in the acute stage of the infection (55–57). The effectiveness of the response of Th1 determines the severity of the disease, and patients with moderate and mild COVID-19 had a high frequency of IFNγ-producing Th1 cells (58, 59). Assumingly, an increase in peripheral blood Th1 in acute COVID-19 is associated with the initiation of an antiviral immune response. Similarly, in chronic pulmonary sarcoidosis, type 1 immune response is also the leading one (58, 60). We have previously shown that patients with chronic sarcoidosis had a decreased amount of Th1 in the peripheral blood (61). Common belief is that these cells migrate to the lungs to maintain foci of chronic inflammation, presented as granulomas. Indeed, increased Th1 frequencies were noted in the BALF of patients with pulmonary sarcoidosis (62). Thus, in pulmonary sarcoidosis, Th1 cells may migrate to the site of inflammation, therefore, their concentration in the peripheral blood decreases. However, we found that after acute COVID-19, the percentage and absolute amount of Th1 were also increased vs. COVID-19 naive patients with sarcoidosis (**Figure 3**). This may be due to increased polarization of Th1 in the periphery during acute COVID-19 infection. Also, given the similar type of immune response, so-called ‘immune interference’ in the effectiveness of immune response due to activation of type 1 response may be observed.

Alongside the Th1 and Th2 balance, research into the pathogenesis of sarcoidosis emphasizes the significance of Th17 cells and their specific subsets. In our analysis of the overall pool of CM CD4+ T cells that can continuously ‘patrol’ secondary lymphoid organs, we observed a reduction in the percentage of CXCR5–CCR6+ Th17 cells in pC Sarc patients compared to HCs (**Figure 4**). Additionally, EM CD4+ T cells (that exit the bloodstream and invade inflamed tissues) were not observed in pC Sarc patients, likely because these cells are specifically migrating to inflammatory sites. The behaviour of Th17 cells in peripheral blood can be complex, as some studies indicate that memory CCR6+ T cells are more abundant in sarcoidosis groups (63), whereas in some studies IL-17A-producing cells numbers are low in peripheral blood (64). Furthermore, both BALF and granuloma tissues demonstrate increase in Th17 cytokine-producing cells (65). As previously discussed, Th17 dynamics in acute COVID-19 are arguable (reviewed in (66)). In patients recovering from COVID-19, Th17 levels can be high for a long time (67). Similar results were seen in the study by Orologas-Stavrou et al., who associate high levels of Th17 with a prolonged pro-inflammatory response in these patients (68). Although longer observations (more then 8 months after recovery) demonstrated no statistical differences in circulating Th17 levels (69).

When studying Th17 subsets among CM CD4+ T cells, we noted an increase of ‘classical’ Th17 cells, whereas the Th17.1 percentage demonstrated a decline in both groups compared to controls (**Table 2**). It was suggested that DN Th17 and DP Th17 represent an early transitional stage of Th17 differentiation (70). While ‘classical’ Th17 cells express RORγt, effectively secrete high concentrations of IL-17 and low concentrations of IFN-γ, ‘non-classical’ Th17.1 cells co-express T‐bet and RORγt, and produce low amounts of IL-17, but high levels of IFN-γ and GM-CSF (71, 72). Both an increase (51) and a decrease (73) in CD4+CCR6+CXCR3+ cells can be seen in the peripheral blood of patients with chronic lung sarcoidosis. ‘Non-classical’ Th17.1 cells are considered to be the main IFNγ-producer cells during granuloma formation (74, 75). It is worth mentioning that in the BALF derived from patients with sarcoidosis, an increase of ligands for CXCR3 and CCR6 (e.g., CXCL10 and CCL20, respectively) is seen (76, 77). As these receptors are present on Th17.1 cells, we can presume that Th17.1 cells can more effectively migrate along the concentration gradient and accumulate in inflammatory sites. Immunohistochemical analysis of sarcoid granulomas revealed increased levels of Th17.1 cells both in the centre and on the periphery of granulomas (51, 78), possibly indicating Th17.1 involvement in autoimmune inflammation. As Th17.1 in patients with sarcoidosis migrate to the inflammatory sites, in acute COVID-19 Th17.1 cells lowered in peripheral blood (38, 55). This decline was observed in CM and EM cells, likely because these cells migrate to sites of inflammation. In another study, the proportion of Th17 cells in the blood of COVID-19 patients increased, while Th17.1 levels remained similar to those of the control group (56). Thus, we can assume that in COVID-19 convalescent patients with sarcoidosis, a decrease in the levels of Th17.1 can be associated with the migration of these cells into the lung tissue to maintain sarcoid granulomas. Additionally, Th17.1 cells were observed in the BALF of patients with sarcoidosis and were associated with disease progression (79). Moreover, history of COVID-19 could contribute to the formation and activation of self-reactive Th17.1, as well as increase their chemotaxis into the lung tissue. However, further investigations are required to better understand the role of ‘classical’ Th17 cells and Th17.1 cells in progression of sarcoidosis in post-COVID-19 patients.

The Tfh cells’ role in sarcoidosis is a relatively new topic. Current study demonstrates that there is an elevated presence of CXCR5+ CM Th cells in individuals with sarcoidosis (**Figure 4**). This finding is supported by data from d’Alesandro et al., which highlights the increased levels of CXCR5+CD45RA– Th cells in sarcoidosis patients compared to control subjects (80). Interstingly, we did observe no changes in EM Tfh cells, that were capable of exiting the bloodstream and migrating to inflamed peripheral tissues, while other research indicated an increase in CXCR3+ Tfh cells in BALF (81). Additionally, study by d’Alessandro et al. have shown increased expression of CD103 on Tfh cells in peripheral blood, BALF, and tissue samples from chest lymph nodes (82). While Ly et al. confirmed elevated levels of CD4+CXCR5+ T cells in skin lesions in sarcoidosis (83). All these data potentially prove the theory on Tfh cells migration into inflammatory cite.

Circulating Tfh cells are diverse and comprise at least four main subsets with distinct functions, while an imbalance among Tfh1 cells, that are responsible for regulatory functions and restriction of humoral immune responses, and Tfh2 and Tfh17 cells, that may support the survival of activated ‘naïve’ B cells, their differentiation into plasma cells and class switch recombination, is linked to pathological alterations in humoral immunity (84–86). Such alteration in Tfh subsets have been observed in various infections and autoimmune diseases (87, 88). Currently, among CM Tfh cells (**Table 3**), we observed an increase in Tfh2 cells in patients with sarcoidosis. Those with a history of COVID-19 showed the most significant increase among the three groups, although DP Tfh cells were at their lowest in this cohort. Additionally, when examining EM Tfh cells (**Table 3**), we found a decrease in Tfh1 cells alongside an increase in Tfh2 and Tfh17 cells in both sarcoidosis patient groups. Preliminary investigations indicated an increase in CM CXCR3–CCR6– Tfh2-like cells in the circulation of patients with chronic sarcoidosis (89). In the paper by Zhou et al., an increase in Tfh2 and Tfh17 cells was seen, whereas Tfh1 and Tfh17.1 demonstrated a decrease in patients with sarcoidosis (90). Another study, however, demonstrated an increase in Tfh1 in peripheral blood and a decrease of Tfh1 and Tfh2 in BALF of patients with chronic sarcoidosis (80). It is possible that the imbalance in pro- and anti-inflammatory Tfh cell subsets can be responsible for autoimmune reactions development, especially in patients with COVID-19 history.

In patients recovering from COVID-19, an imbalance in circulating Tfh cells was also seen. Specifically, there was an increase in CXCR5+CD45RA– memory T cells among COVID-19 convalescents (91). Those who had successfully recovered showed elevated levels of Tfh2, Tfh17, and DP Tfh cells compared to control groups. Previous research indicated that severe infections were associated with the highest percentages of Tfh1 cells, which correlated with neutralizing antibody titers (92). Other studies noted an increase in circulating Tfh1 and Tfh2 cells, while Tfh17 levels were significantly lower (93). Over a period of 6-7 months of monitoring patients recovering from COVID-19, Tfh1 cell levels returned to normal ranges, whereas initially high levels of Tfh2 decreased (94). Additionally, some studies have suggested that the increased activity of Tfh cells in convalescents could potentially lead to autoimmune and allergic reactions leading to long COVID-19 (93, 95). Thus, acute COVID-19 infection and sarcoidosis both found to alter Tfh immune response, and, in general, can have a dramatic effect on B cells and humoral adaptive immunity. Therefore, a decrease in Tfh1 in the peripheral blood of patients with sarcoidosis and COVID-19 convalescents with sarcoidosis may indicate a decrease in the regulatory control of B cells and a violation of the affinity of formed antibodies in response to an antigen. Moreover, a simultaneous increase in Tfh2 and Tfh17 may characterize a switch in the class of antibodies, with a simultaneous violation of regulation by Tfh1, which can contribute to autoimmune reactions.

## 5 Conclusions

The underlying mechanisms of sarcoidosis development after COVID-19 are not yet understood, and any research on the topic is still very rare. Our data demonstrate, that patients with sarcoidosis and history of COVID-19 demonstrate a T cell imbalance, characterized by alterations in regulatory T cells and various effector T helper subsets, including Th1, Th2, Th17 and Tfh cell subsets (summarized in **Supplementary Figure 8**, volcano plot A). These alterations may contribute to the clinical progression of chronic lung sarcoidosis and the development of fibrosis. Interestingly, the dynamics of Th cell subsets in COVID-19 and sarcoidosis have a number of similarities (87, 96, 97). Thus, after COVID-19 an increase in Th cell subsets in the lung, the process of granuloma formation can begin. Furthermore, there is a similar imbalance in Tfh cells associated with the alterations in adaptive humoral immune response. Tfh cells and B cells after coronavirus infection may be capable of triggering the formation of sarcoid granulomas in the lung tissue due to a failure of tolerance and initiation of autoimmune reactions, indicating the importance of B and T cells cross-talk in the pathogenesis of sarcoidosis after acute COVID-19. Finally, the complex interaction between sarcoidosis and COVID-19 remains unclear, and long-term observations on COVID-19 convalescent patients with sarcoidosis are needed to consolidate our findings and literary data. Collectively, the evidence underscores the importance of detailed immune profiling in sarcoidosis, especially in the post-COVID-19 era, to understand the mechanistic underpinnings of disease modulation and to identify potential therapeutic targets.

## 6 Limitations of the study

Despite the significance of the findings, this study has several limitations that should be considered when interpreting the results. The relatively small sample size may affect the statistical power and limit the generalizability of the conclusions to a broader population of patients with sarcoidosis and prior COVID-19 infection. Furthermore, functional activity of the identified T-cell subsets was not assessed, which complicates interpretation of their role in the pathogenesis of sarcoidosis following COVID-19. Potential confounding effects of therapeutic interventions, such as anti-inflammatory and immunomodulatory treatments used by patients, were not fully accounted for in the analysis.

It should also be noted that evaluation of CD4+ T-cell subpopulations was performed using peripheral blood samples, which may not fully reflect immune activity within affected tissues, such as the lungs or lymph nodes, where granuloma formation occurs. More comprehensive understanding of the immunopathogenesis requires further studies including tissue biopsy analysis and functional assays.

This study employed a cross-sectional design without access to pre-infection baseline samples or longitudinal follow-up. Thus, we cannot determine whether the observed immunophenotypic changes are persistent, transient, or pre-existing. Future prospective studies with serial sampling are required to clarify these dynamics.

The lack of a COVID-19 positive control group without sarcoidosis limits the ability to attribute the observed T cell changes specifically to the interaction between SARS-CoV-2 and granulomatous inflammation. Future studies incorporating matched post-COVID controls without sarcoidosis are warranted. Furthermore, patient groups I and II included only patients with pulmonary sarcoidosis.

## Data Availability

The original contributions presented in the study are included in the article/**Supplementary Material**. Further inquiries can be directed to the corresponding author.

## Supplementary material

Supplementary Table 1 List of monoclonal antibodies for immunophenotyping of peripheral blood maturation and ‘polarized’ CD4+ T cell subsets (CD25, CD4, CD8 were manufactured by Beckman Coulter, Indianapolis, IN, USA, and CD183, CD185, CD194, CD196, CD3, CD197, CD45RA were manufactured by BioLegend, Inc., San Diego, CA, USA);

Supplementary Figure 1 Gating and analysis strategy for maturation and ‘polarized’ CD4+ T cell subsets immunophenotyping using 10-color flow cytometry;

Supplementary Figure 2 Alterations in frequencies of circulating CD4+ T cell maturation subsets in patients with sarcoidosis and COVID-19 convalescent patients with sarcoidosis;

Supplementary Figure 3 Alterations in frequencies of ‘polarized’ CD4+ T cell subsets in patients with sarcoidosis and COVID-19 convalescent patients with sarcoidosis;

Supplementary Figure 4 Imbalance of ‘polarized’ Th cell subsets within central memory Th cell compartments in patients with sarcoidosis and COVID-19 convalescent patients with sarcoidosis;

Supplementary Figure 5 Imbalance of ‘polarized’ Th cell subsets within effector memory Th cell compartments in patients with sarcoidosis and COVID-19 convalescent patients with sarcoidosis;

Supplementary Figure 6 Memory Th17 cell subsets in patients with sarcoidosis and COVID-19 convalescent patients with sarcoidosis;

Supplementary Figure 7 Memory follicular Th cell subsets in patients with sarcoidosis and COVID-19 convalescent patients with sarcoidosis;

Supplementary Figure 8 Peripheral blood T cell subsets changes in patients with sarcoidosis and COVID-19 convalescent patients with sarcoidosis.

## Glossary

ACE2: angiotensin-converting enzyme 2
ATP: adenosine triphosphate
BALF: bronchoalveolar lavage fluid
CCL17: C−C motif chemokine ligand 17
CCL18: C−C motif chemokine ligand 18
CCL22: C−C motif chemokine ligand 22
CM: central memory
COVID-19: Coronavirus Disease-2019
CT: computed tomography
CXCL10: CXC motif chemokine ligand 10
DN: double negative
DP: double positive
DPP4: dipeptidyl-peptidase 4
EM: effector memory
GATA3: GATA binding protein 3
GM-CSF: Granulocyte–macrophage colony stimulating factor
HC: healthy controls
IFNg: interferon gamma
IL: Interleukin
IL4R: Interleukin-4 receptor
LCR: long-time clinically recovered
MBT: Mycobacterium tuberculosis
MDC: Macrophage-derived chemokine
PBMC: peripheral blood mononuclear cell
pC Sarc: COVID-19 convalescent patients with sarcoidosis
PCR: polymerase chain reaction
RORγt: RAR-related orphan receptor gamma
Sarc: COVID-19 naïve patients with sarcoidosis
SARS-CoV-2: severe acute respiratory syndrome coronavirus 2
SCR: short-time clinically recovered
TARC: thymus and activation-regulated chemokine
T-bet: T-box expressed in T cells
TEMRA: effector memory cells re-expressing CD45RA
Tfh: follicular Th cells
TGFβ: Transforming growth factor-β
Th: T helper cell
TREC: T-cell recombination excision circle
Tregs: regulatory T cells
tSNE: t-Distributed Stochastic Neighbor Embedding
