# Supplementary material for: Immune responses in pulmonary sarcoidosis following COVID-19
Source: Front Immunol. 2025 Nov 25;16:1614461. doi: 10.3389/fimmu.2025.1614461 (PMC12685880; doi:10.3389/fimmu.2025.1614461)
Supplement: Supplementary Table 1 — List of monoclonal antibodies for immunophenotyping of peripheral blood maturation and ‘polarized’ CD4+ T cell subsets (CD25, CD4, CD8 were manufactured by Beckman Coulter, Indianapolis, IN, USA, and CD183, CD185, CD194, CD196, CD3, CD197, CD45RA were manufactured by BioLegend, Inc., San Diego, CA, USA); [file Table1.docx]

Supplementary Material

**Supplementary Table 1.** List of monoclonal antibodies for immunophenotyping of peripheral blood maturation and ‘polarized’ CD4+ T cell subsets (CD25, CD4, CD8 were manufactured by Beckman Coulter, Indianapolis, IN, USA, and CD183, CD185, CD194, CD196, CD3, CD197, CD45RA were manufactured by BioLegend, Inc., San Diego, CA, USA).

| **N** | **Antigen** | **Fluorochome** | **Clone** | **Isotype** | **Cat. number** |
| --- | --- | --- | --- | --- | --- |
| **1** | CD183 (CXCR3) | Alexa Fluor 488 | G025H7 | Mouse IgG1, k | 353710 |
| **2** | CD25 | PE | B1.49.9 | IgG2a Mouse | A07774 |
| **3** | CD185 (CXCR5) | PE/Dazzle™ 594 | J252D4 | Mouse IgG1, k | 356928 |
| **4** | CD194 (CCR4) | PerCP/Cy5.5 | L291H4 | Mouse IgG1, k | 359406 |
| **5** | CD196 (CCR6) | PE/Cy7 | G034E3 | Mouse IgG2b, k | 353418 |
| **6** | CD4 | APC | 13B8.2 | IgG1 Mouse | IM2468 |
| **7** | CD8 | APC-AF700 | B9.11 | IgG1 Mouse | B49181 |
| **8** | CD3 | APC/Cy7 | HIT3a | Mouse IgG2a, k | 300318 |
| **9** | CD197 (CCR7) | Brilliant Violet 421 | G043H7 | Mouse IgG2a, k | 353208 |
| **10** | CD45RA | Brilliant Violet 510 | HI100 | Mouse IgG2b, k | 304142 |

**
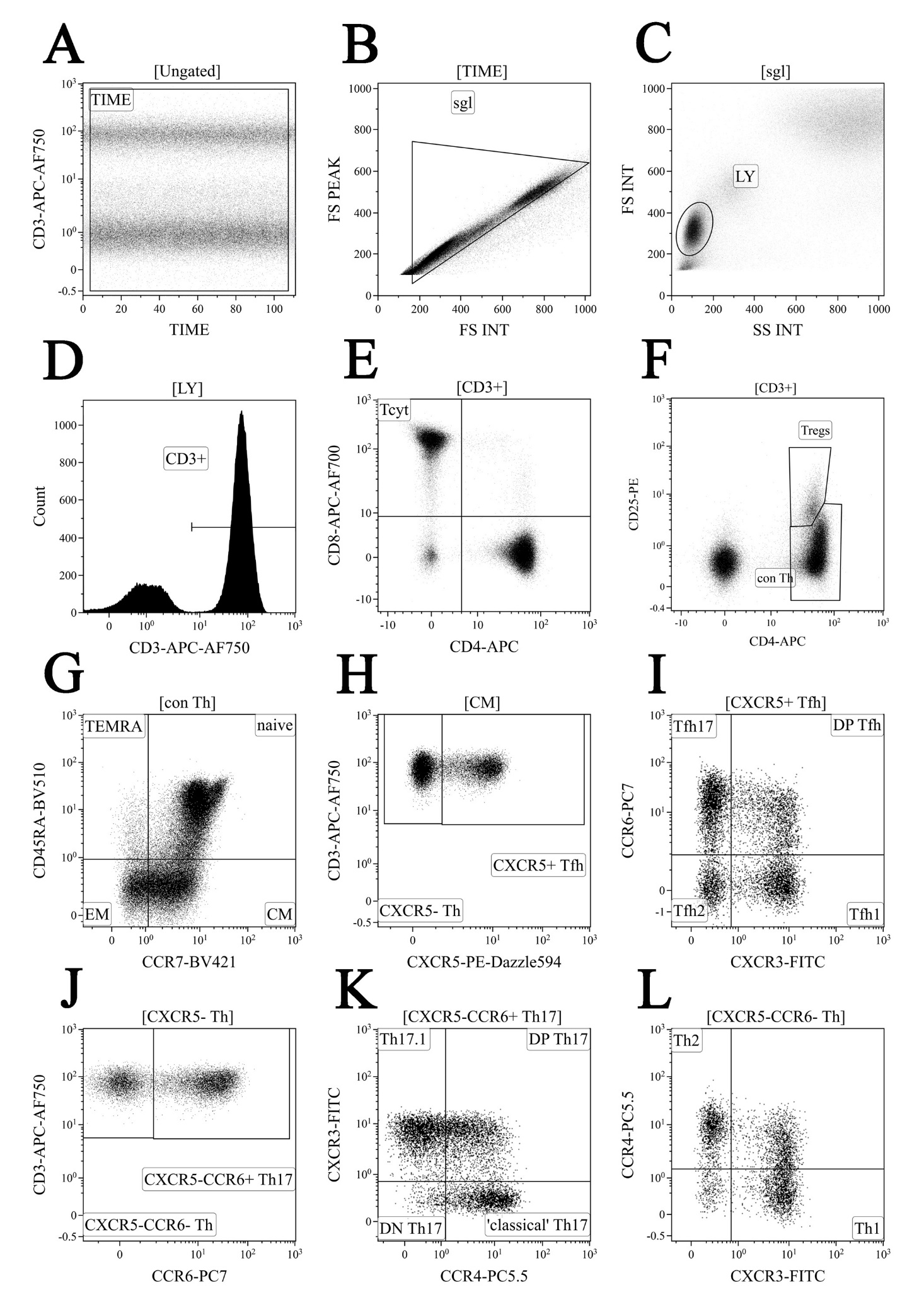
**

**Supplementary Figure 1.** Gating and analysis strategy for maturation and ‘polarized’ CD4+ T cell subsets immunophenotyping using 10-color flow cytometry. Dot plot (A) – artifact exclusion included time gating; dot plot (B) – doublets exclusion from the analysis using the ratio between integral and peak forward scatter signals; dot plot (C) – total lymphocyte subset purification based on side scatter and forward scatter; dot plot (D) – total T cell subset gaiting based on CD3 expression; dot plot (E) – detection of CD8+ T cells (CD3+CD8+) within total CD3+ T cell subset; dot plot (F) – identification regulatory T cells (CD3+CD4+CD25bright) and conventional Th cells; dot plot (G) – four main CD4+ T cell maturation subsets were identified, including ‘naïve’ cells (CD45RA+CCR7+), central and effector memory cells (CD45RA–CCR7+ and CD45RA–CCR7–, respectively), as well as effector memory CD45RA-positive Th cells (TEMRA) with CD45RA+CCR7– phenotype. Next, within total CD3+CD4+ Th cell compartment (shown as an example) Th lineages was categorized into 4 functional subsets, including CXCR5+ follicular Th cells (Tfh) (dot plot H), CXCR5–CCR6+ Th17 cells (dot plot J), and CXCR5–CCR6–CXCR3+CCR4– Th1 and CXCR5–CCR6–CXCR3–CCR4+ Th2 cells (dot plot L). Dot plot I – four distinct subsets of follicular Th cells were identified, including Tfh1 (CXCR3+CCR6–), Tfh17 (CXCR3–CCR6+), Tfh2 (CXCR3–CCR6–), and ‘double positive’ DP Tfh (CXCR3+CCR6+). Dot plot K – total CXCR5–CCR6+ Th17 subsets was subdivided into ‘classical’ CCR6+CCR4+ Th17 cells, ‘non-classical’ CCR6+CXCR3+ Th17.1, and lacking (double negative, DN Th17) or co-expressing CXCR3 and CCR4 (double positive, DP Th17). Furthermore, all ‘polarized’ Th cell subsets were also identified within central and effector memory cells with CD45RA–CCR7+ and CD45RA–CCR7– phenotypes.


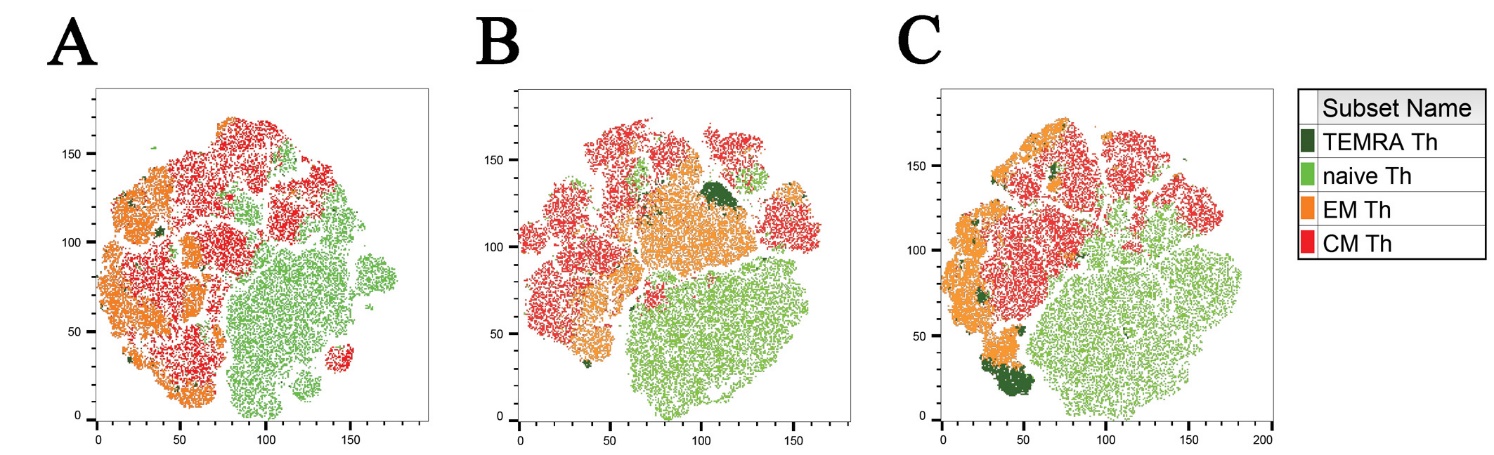


**Supplementary Figure 2.** Alterations in frequencies of circulating CD4+ T cell maturation subsets in patients with sarcoidosis and COVID-19 convalescent patients with sarcoidosis.

t-Distributed Stochastic Neighbor Embedding (tSNE) data A – tSNE of concatenated data of 6 patients with pulmonary sarcoidosis; tSNE data B - tSNE of concatenated data of 6 COVID-19 convalescent patients with pulmonary sarcoidosis; tSNE data C – tSNE of concatenated data of 6 healthy controls. Co-expression of CD45RA and CCR7 was analyzed on Th cells, four maturation subsets of Th cells were identified, including ‘naïve” cells (CD45RA+CCR7+), central and effector memory cells (CD45RA–CCR7+ and CD45RA–CCR7–, respectively), as well as TEMRA Th cells with CD45RA+CCR7– phenotype. Surface marker distribution is depicted in the tSNE map. Relative antigen expression is visualized by the color tone (as it shown in the upper right part of the Figure 2).

**
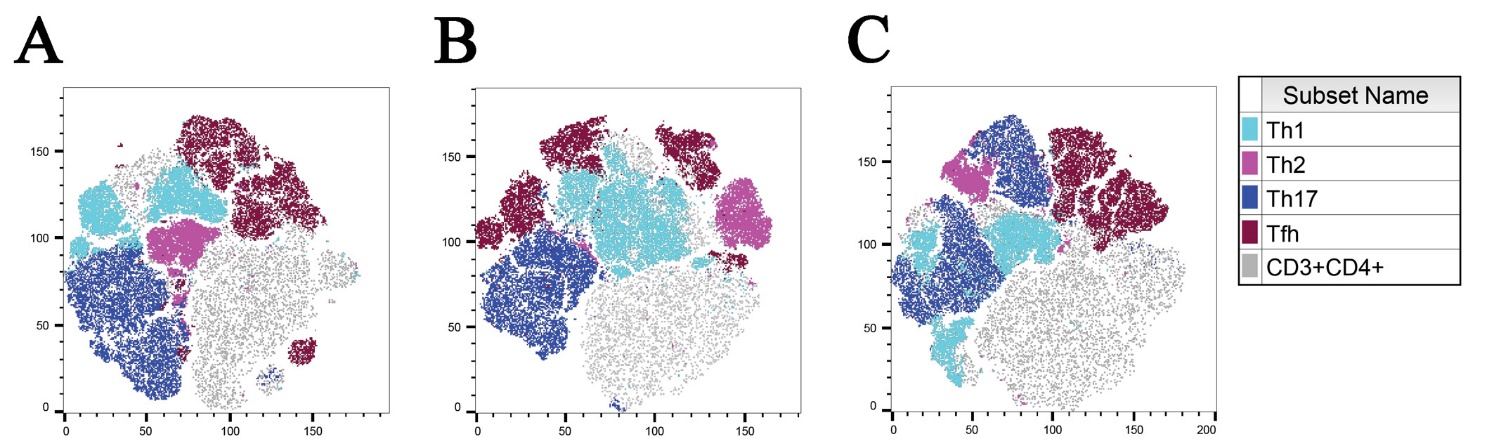
**

**Supplementary Figure 3.** Alterations in frequencies of ‘polarized’ CD4+ T cell subsets in patients with sarcoidosis and COVID-19 convalescent patients with sarcoidosis.

tSNE data A – tSNE of concatenated data of 6 patients with pulmonary sarcoidosis; tSNE data B - tSNE of concatenated data of 6 COVID-19 convalescent patients with pulmonary sarcoidosis; tSNE data C – tSNE of concatenated data of 6 healthy controls. Within total CD3+CD4+ Th cell compartment Th lineages was categorized into 4 functional subsets, including CXCR5– follicular Th cells (Tfh), CXCR5–CCR6+ Th17 cells, and CXCR5–CCR6–CXCR3+CCR4– Th1 and CXCR5–CCR6–CXCR3–CCR4+ Th2 cells. Surface marker distribution is depicted in the tSNE map. Relative antigen expression is visualized by the color tone (as it shown in the upper right part of the Figure 3).


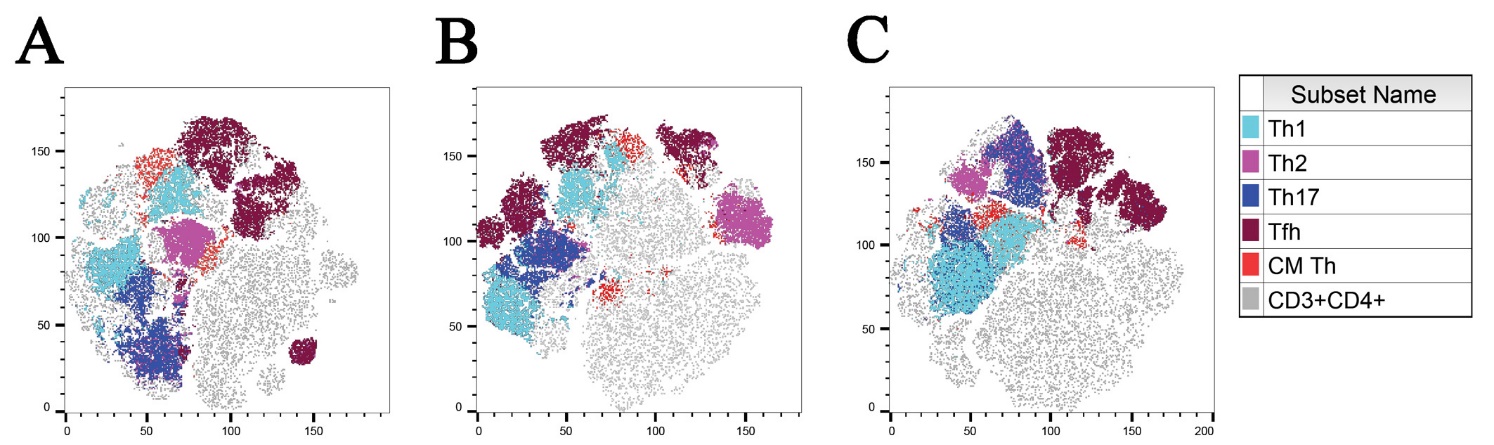


**Supplementary Figure 4.** Imbalance of ‘polarized’ Th cell subsets within central memory Th cell compartments in patients with sarcoidosis and COVID-19 convalescent patients with sarcoidosis.

tSNE data A – tSNE of concatenated data of 6 patients with pulmonary sarcoidosis; tSNE data B - tSNE of concatenated data of 6 COVID-19 convalescent patients with pulmonary sarcoidosis; tSNE data C – tSNE of concatenated data of 6 healthy controls. Within central memory CD3+CD4+CD45RA–CCR7+ Th cell compartment Th lineages was categorized into 4 functional subsets, including CXCR5– follicular Th cells (Tfh), CXCR5–CCR6+ Th17 cells, and CXCR5–CCR6–CXCR3+CCR4– Th1 and CXCR5–CCR6–CXCR3–CCR4+ Th2 cells. Surface marker distribution is depicted in the tSNE map. Relative antigen expression is visualized by the color tone (as it shown in the upper right part of the Figure 4).


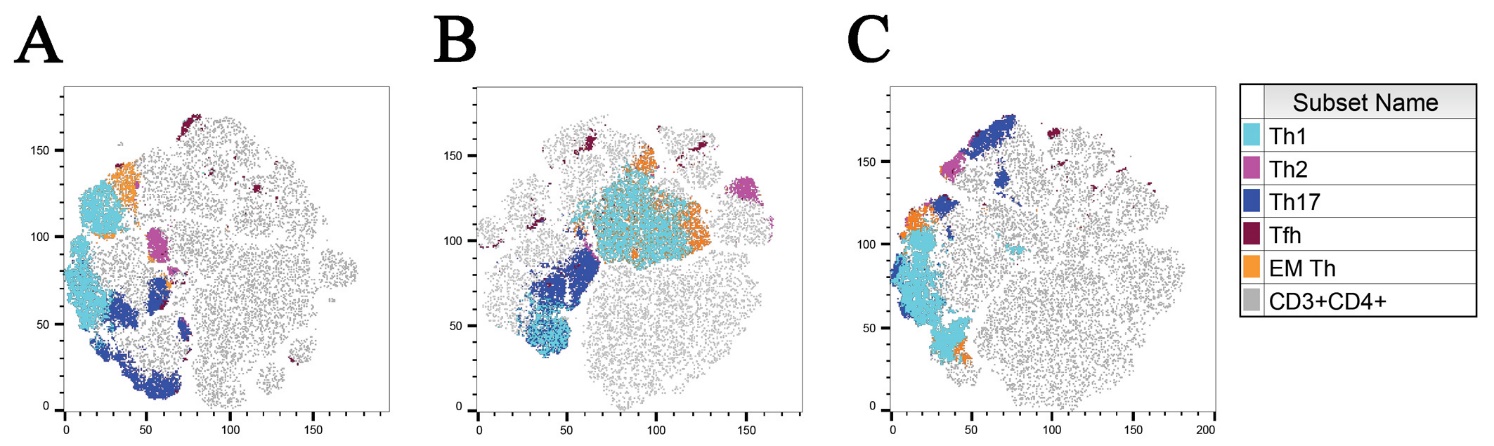


**Supplementary Figure 5.** Imbalance of ‘polarized’ Th cell subsets within effector memory Th cell compartments in patients with sarcoidosis and COVID-19 convalescent patients with sarcoidosis.

tSNE data A – tSNE of concatenated data of 6 patients with pulmonary sarcoidosis; tSNE data B - tSNE of concatenated data of 6 COVID-19 convalescent patients with pulmonary sarcoidosis; tSNE data C – tSNE of concatenated data of 6 healthy controls. Within effector memory CD3+CD4+CD45RA–CCR7– Th cell compartment Th lineages was categorized into 4 functional subsets, including CXCR5– follicular Th cells (Tfh), CXCR5–CCR6+ Th17 cells, and CXCR5–CCR6–CXCR3+CCR4– Th1 and CXCR5–CCR6–CXCR3–CCR4+ Th2 cells. Surface marker distribution is depicted in the tSNE map. Relative antigen expression is visualized by the color tone (as it shown in the upper right part of the Figure 5).

**
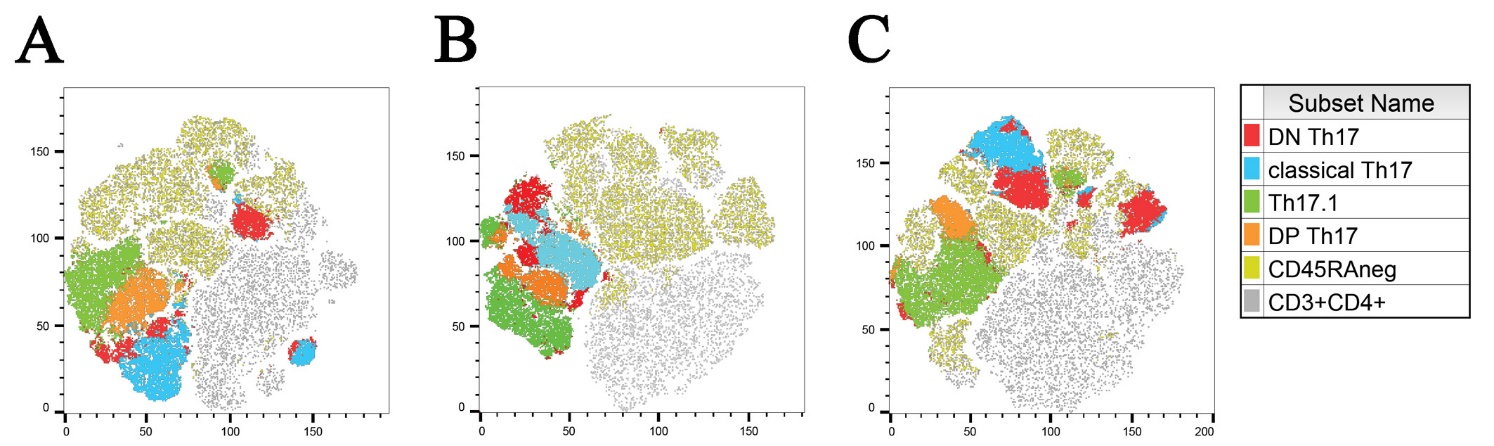
**

**Supplementary Figure 6.** Memory Th17 cell subsets in patients with sarcoidosis and COVID-19 convalescent patients with sarcoidosis.

tSNE data A – tSNE of concatenated data of 6 patients with pulmonary sarcoidosis; tSNE data B - tSNE of concatenated data of 6 COVID-19 convalescent patients with pulmonary sarcoidosis; tSNE data C – tSNE of concatenated data of 6 healthy controls. Within total memory CD3+CD4+CD45RA–CCR7+CXCR5+ follicular Th cells four distinct subtypes of Tfh were identified, including Tfh1 (CXCR3+CCR6–), Tfh17 (CXCR3–CCR6+), Tfh2 (CXCR3–CCR6–), and DP Tfh (CXCR3+CCR6+). Surface marker distribution is depicted in the tSNE map. Relative antigen expression is visualized by the color tone (as it shown in the upper right part of the Figure 6).

**
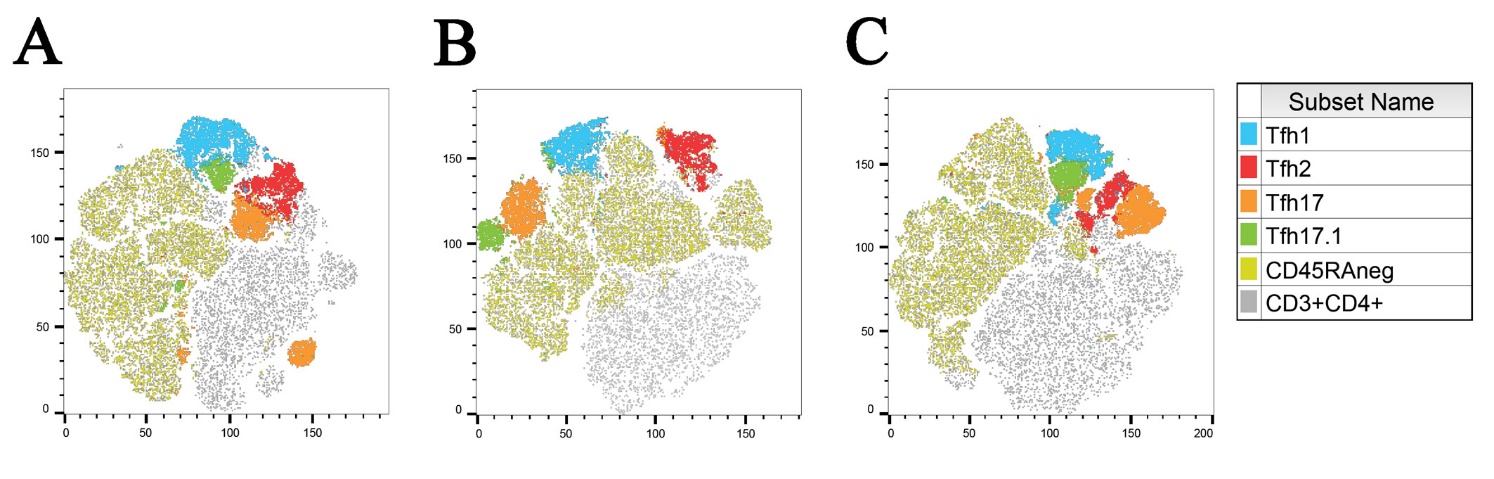
**

**Supplementary Figure 7.** Memory follicular Th cell subsets in patients with sarcoidosis and COVID-19 convalescent patients with sarcoidosis.

tSNE data A – tSNE of concatenated data of 6 patients with pulmonary sarcoidosis; tSNE data B - tSNE of concatenated data of 6 COVID-19 convalescent patients with pulmonary sarcoidosis; tSNE data C – tSNE of concatenated data of 6 healthy controls. Total memory CD3+CD4+CD45RA–CXCR5+ Tfh cells were subdivide into CXCR3+CCR6– Tfh1 cells, CXCR3–CCR6– Tfh2 cells, CXCR3–CCR6+ Tfh17cells and CXCR3+CCR6+ Tfh17.1 cells. Surface marker distribution is depicted in the tSNE map. Relative antigen expression is visualized by the color tone (as it shown in the upper right part of the Figure 7).

**
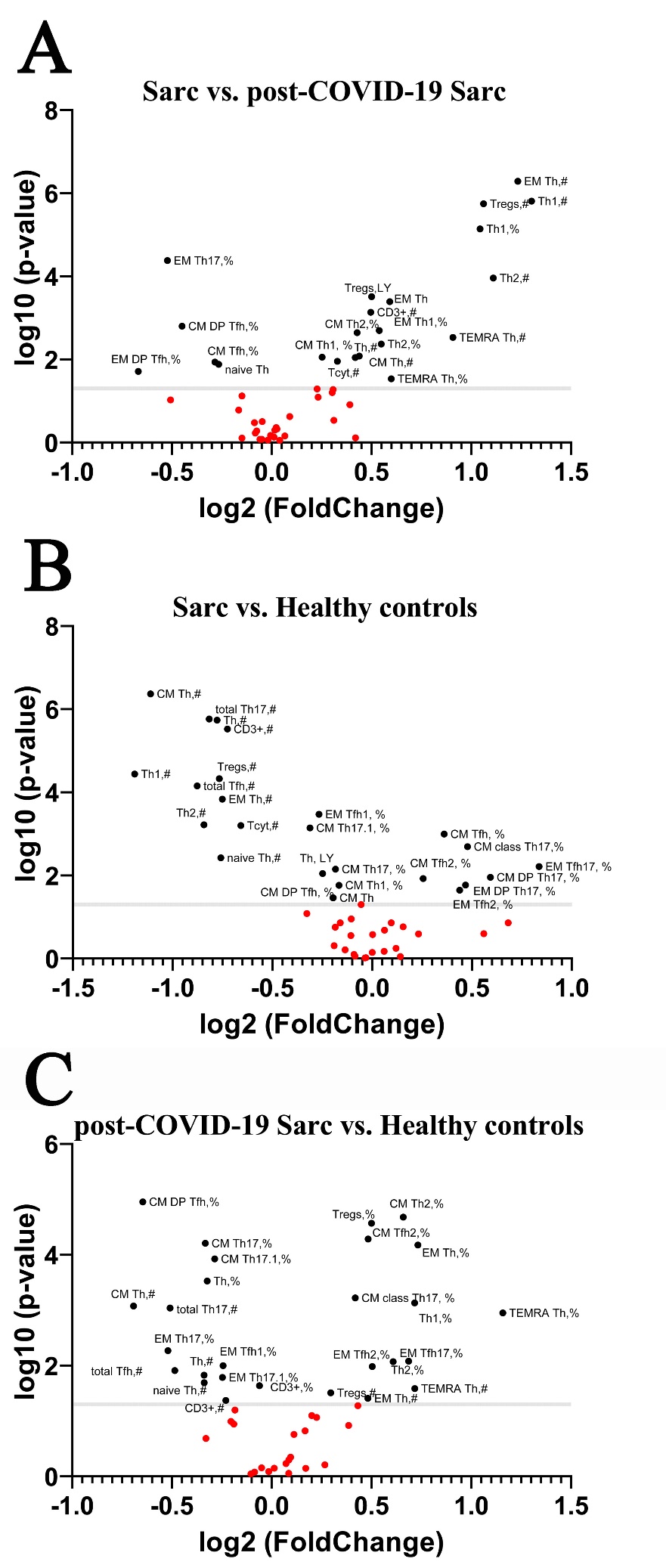
**

**Supplementary Figure 8.** Peripheral blood T cell subsets changes in patients with sarcoidosis and COVID-19 convalescent patients with sarcoidosis.

Volcano plot A illustrating the 48 ranked CD3+ T cell subsets count changes between patient with sarcoidosis and COVID-19 convalescent patient with sarcoidosis; volcano plot B illustrating the 48 ranked CD3+ T cell subsets count changes between patient with sarcoidosis and healthy controls; volcano plot C illustrating the 48 ranked CD3+ T cell subsets count changes between COVID-19 convalescent patient with sarcoidosis and healthy controls. The −log10 corrected p-value is plotted against the log2 fold change. Red coloring points below the line denotes p > 0.05, which is our significance threshold (prior to logarithmic transformation). Samples were from patients with sarcoidosis without SARS-CoV-2 infection (n = 30), COVID-19 convalescent patients with sarcoidosis (n = 31), and healthy controls (n = 40). Statistical significance was assessed using Mann–Whitney U-test.
